# Modern work-up and extended resection in perihilar cholangiocarcinoma: the AMC experience

**DOI:** 10.1007/s00423-018-1649-2

**Published:** 2018-01-19

**Authors:** F. Rassam, E. Roos, K. P. van Lienden, J. E. van Hooft, H. J. Klümpen, G. van Tienhoven, R. J. Bennink, M. R. Engelbrecht, A. Schoorlemmer, U. H. W. Beuers, J. Verheij, M. G. Besselink, O. R. Busch, T. M. van Gulik

**Affiliations:** 1 Department of Surgery, Academic Medical Center, Amsterdam, The Netherlands; 2 Department of Radiology and Nuclear Medicine, Academic Medical Center, Amsterdam, The Netherlands; 3 Department of Gastroenterology & Hepatology and Tytgat Institute for Liver and Intestinal Research, Academic Medical Center, Amsterdam, The Netherlands; 4 Department of Medical Oncology, Academic Medical Center, Amsterdam, The Netherlands; 5 Department of Radiotherapy, Academic Medical Center, Amsterdam, The Netherlands; 6 Department of Pathology, Academic Medical Center, Amsterdam, The Netherlands

**Keywords:** Perihilar cholangiocarcinoma, Klatskin tumor, Diagnosis, Staging, Biomarkers, Preoperative assessment, Hepato-biliary scintigraphy, Biliary drainage, Surgical resection, Postoperative outcome

## Abstract

**Aim:**

Perihilar cholangiocarcinoma (PHC) is a challenging disease and requires aggressive surgical treatment in order to achieve curation. The assessment and work-up of patients with presumed PHC is multidisciplinary, complex and requires extensive experience. The aim of this paper is to review current aspects of diagnosis, preoperative work-up and extended resection in patients with PHC from the perspective of our own institutional experience with this complex tumor.

**Methods:**

We provided a review of applied modalities in the diagnosis and work-up of PHC according to current literature. All patients with presumed PHC in our center between 2000 and 2016 were identified and described. The types of resection, surgical techniques and outcomes were analyzed.

**Results and conclusion:**

Upcoming diagnostic modalities such as Spyglass and combinations of serum biomarkers and molecular markers have potential to decrease the rate of misdiagnosis of benign, inflammatory disease. Assessment of liver function with hepatobiliary scintigraphy provides better information on the future remnant liver (FRL) than volume alone. The selective use of staging laparoscopy is advisable to avoid futile laparotomies. In patients requiring extended resection, selective preoperative biliary drainage is mandatory in cholangitis and when FRL is small (< 50%). Preoperative portal vein embolization (PVE) is used when FRL volume is less than 40% and optionally includes the left portal vein branches to segment 4. Associating liver partition and portal vein ligation for staged hepatectomy (ALPPS) as alternative to PVE is not recommended in PHC. N2 positive lymph nodes preclude long-term survival. The benefit of unconditional *en bloc* resection of the portal vein bifurcation is uncertain. Along these lines, an aggressive surgical approach encompassing extended liver resection including segment 1, regional lymphadenectomy and conditional portal venous resection translates into favorable long-term survival.

## Introduction

Cholangiocarcinoma accounts for 3% of all gastrointestinal malignancies worldwide [1]. The tumors arise from the epithelium of the biliary tract and may occur in the whole biliary ductal system. They are sub-classified according to their location, in intrahepatic, perihilar and distal cholangiocarcinoma [2]. Each entity comes with a specific set of problems and therefore, management requires a tailored approach.

**Fig. 1:**
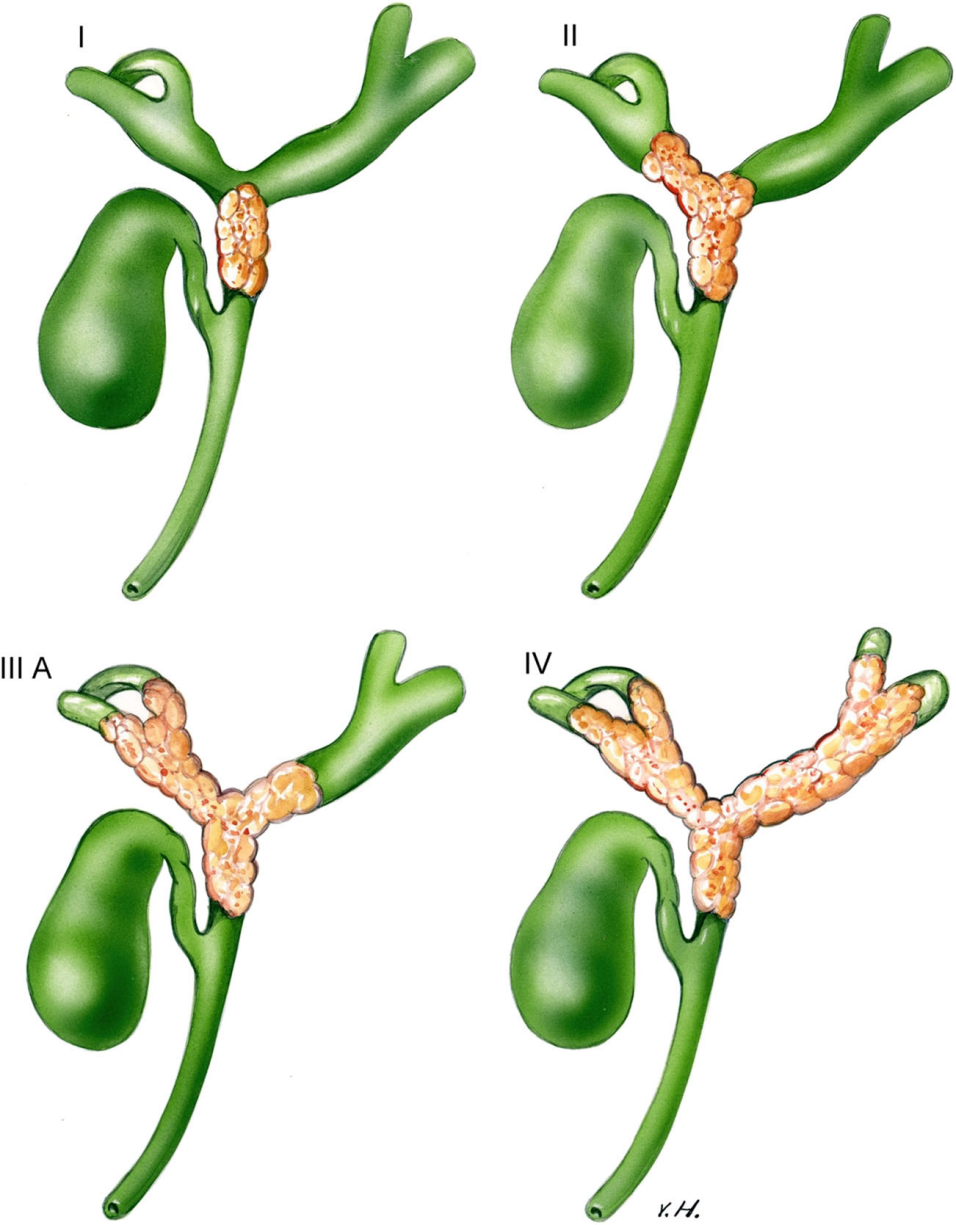
Bismuth-Corlette classification for staging of perihilar cholangiocarcinoma

Perihilar cholangiocarcinoma (PHC), also known as Klatskin tumor, is the most frequent biliary tract tumor and accounts for approximately 60% of all cholangiocarcinoma’s [3]. This tumor originates in the extrahepatic biliary tract proximal to the origin of the cystic duct, up until the second-degree bile ducts. PHC can be subdivided according to proximal extent of the tumor into the bile ducts (Bismuth-Corlette classification) [4] (Fig. 1).

The incidence of cholangiocarcinoma varies widely between regions. In Asian populations and Chili, parasitic infections are strongly associated with PHC, showing a peak incidence in Thailand of 87 per 100,000 [3, 5–7]. In Western populations the incidence is considerably lower, 1–2 per 100,000, and PHC is mainly associated with primary sclerosing cholangitis (PSC) [8, 9].

Early symptoms are not specific and patients typically present with the sequelae of biliary obstruction. When jaundice finally develops due to local biliary obstruction, patients are often not resectable anymore, and thus not curable. Up to 65–80% of patients have initially unresectable disease due to extensive hepatic artery and/or portal vein infiltration by tumor or distant metastases at time of presentation [10–13]. Of all patients who in time undergo a laparotomy, 40–70% ultimately have resectable disease [14–16]. Patients face many obstacles during diagnosis and work-up for extended resection. These problems range from confirmation of malignancy to cholestasis and cholangitis due to biliary obstruction, requiring biliary drainage.

Oncological outcomes depend heavily on the possibility of performing a radical resection. Patients with unresectable disease, receiving palliative chemotherapy with gemcitabine and cisplatin, have an overall median survival of approximately 12 months [17, 18]. In contrast, median survival of patients with an R0 resection is 30–46 months and 5-year survival rates range from 25 to 40% [19, 20]. The aggressive surgical approach necessary to achieve an R0 resection however, is associated with significant postoperative morbidity and mortality with reported morbidity rates ranging from 60 to 70% [21] and mortality rates as high as 5–18% [19, 22–24]. It is therefore crucial to optimize patients before exposing them to this high-risk surgery.

The aim of this review is to elaborate current diagnosis and work-up and to review the issues of extended resection in patients presenting with a hilar lesion suspicious of PHC, from the perspective of the long-standing experience with this complex tumor in our referral center.

### The AMC experience; the denominator of patients referred with (suspected) PHC

Between 2000 and 2016, a total of 606 patients with lesions suspicious of PHC have been referred to our center. Patients were discussed in our HPB oncology multidisciplinary meeting, consisting of experienced hepatobiliary surgeons, dedicated endoscopists, (interventional, abdominal and nuclear) radiologists, radiotherapists, nurse practitioners, medical oncologists and pathologists.

**Fig. 2:**
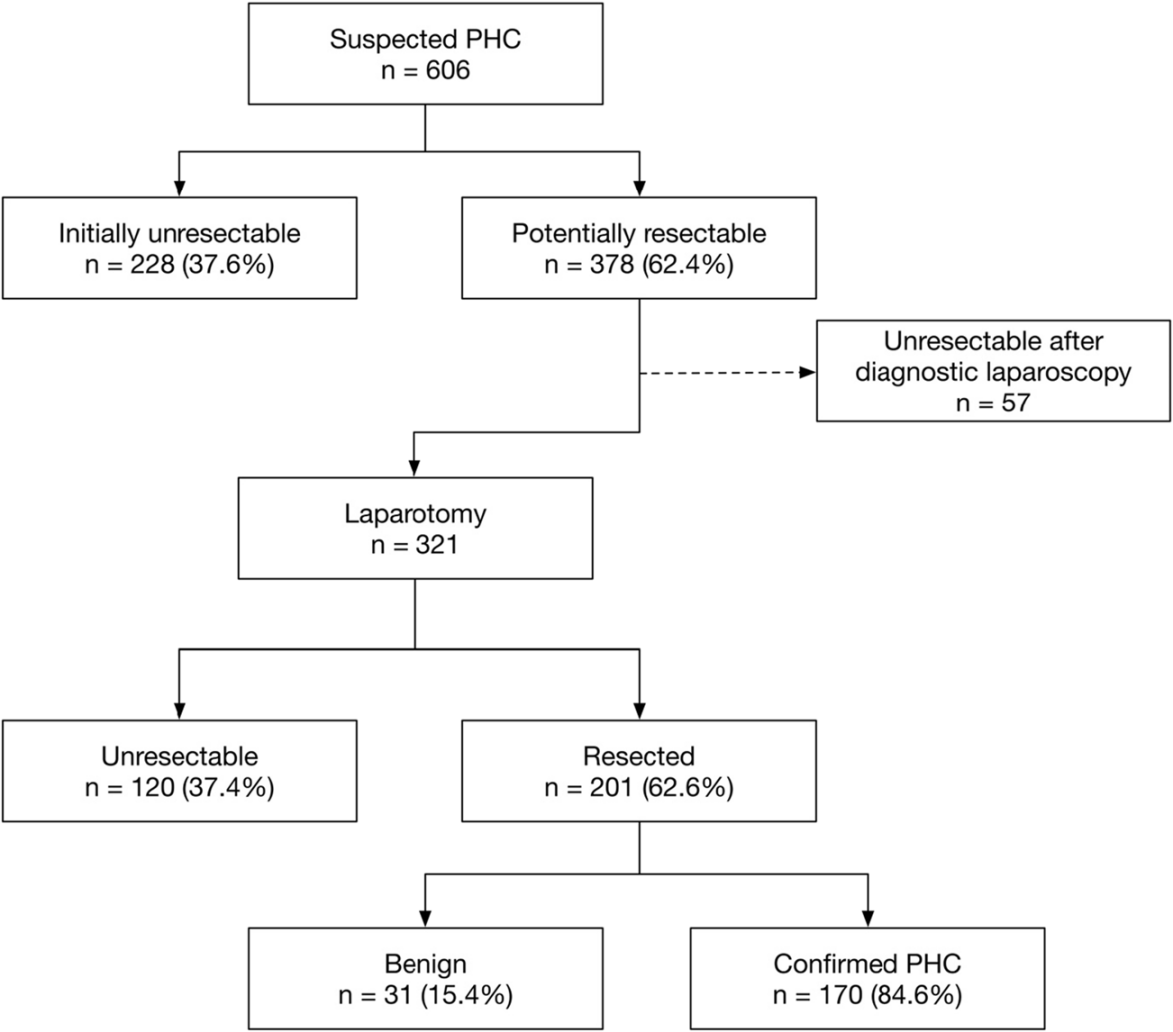
Flow diagram of patients referred to the AMC with suspicion on PHC (2000–2016)

A total of 285 (47.0%) patients were deemed unresectable, of which 228 (37.6%) were found to be unresectable at initial presentation on the basis of imaging studies. The remaining 57 patients were staged with unresectable disease after diagnostic laparoscopy (Fig. 2). The main reason for unresectability was locally advanced disease (*n* = 104), N2 lymph node metastases (*n* = 29), liver metastases (*n* = 27), peritoneal or distant metastases (*n* = 68) or unfitness for major resection (*n* = 53) (Table 1).

**Table 1:** Reasons for unresectability in patients referred with PHC

Reason for unresectability	Patients *n* (%)
Initially unresectable	285
After imaging/laboratory assessment	228
After diagnostic laparoscopy	57
Locally advanced disease	104 (36.5%)
LN metastases	29 (10.2%)
Liver metastases	27 (9.5%)
Peritoneal/distant metastases	68 (23.9%)
Unfit for surgery	53 (18.6%)
Missing	4 (1.4%)
Unresectable during laparotomy	120
Locally advanced disease	43 (35.8%)
LN metastases	39 (32.5%)
Liver metastases	11 (9.2%)
Peritoneal/distant metastases	26 (21.7%)
Unfit for surgery	1 (0.8%)

The remaining 321 (53.0%) patients underwent laparotomy; 120 (19.8%) patients were deemed unresectable on the basis of intraoperative findings. The main reasons were locally advanced disease (*n* = 43), N2 lymph node metastases, (*n* = 39), liver metastases (*n* = 11), peritoneal or other distant metastases (*n* = 26) or major liver resection precluded by comorbidities (*n* = 1) (Table 1).

A total of 201 patients underwent extrahepatic bile duct resection in the majority of cases combined with (extended) liver resection. Of these patients, 66 (32.8%) underwent a left hemihepatectomy, 8 (4.0%) underwent an extended left hemihepatectomy, 31 (15.4%) patients underwent a right hemihepatectomy, 51 (25.4%) underwent an extended right hemihepatectomy, 8 (4.0%) patients underwent resection of 1 or 2 segments and the remainder of 37 (18.4%) patients underwent bile duct resection alone (Table 2).

**Table 2:** Types of resection undertaken in 201 patients with presumed PHC

Type of resection	Patients *n* (%)
Total number of patients	201
Left hemihepatectomy	66 (32.8%)
Right hemihepatectomy	8 (4.0%)
Extended left hemihepatectomy	31 (15.4%)
Extended right hemihepatectomy	51 (25.4%)
Segmentectomy (≤ 3 Couinaud segments)	8 (4.0%)
Only local excision of hilar bile ducts	37 (18.4%)
Including portal vein resection	30/151 (19.9%), 50 missing

Based on pathological examination of the resection specimens, 170 (84.6%) patients had PHC and 31 (15.4%) had benign disease (either unspecified sclerosing cholangitis or IgG4-associated cholangitis) (Fig. 2).

Severe complications (Clavien-Dindo grade 3 or higher) were observed in 93(46.3%) of resected patients. Of all patients who underwent resection, 18 (9.0%) died within the first 90 days.

**Fig. 3:**
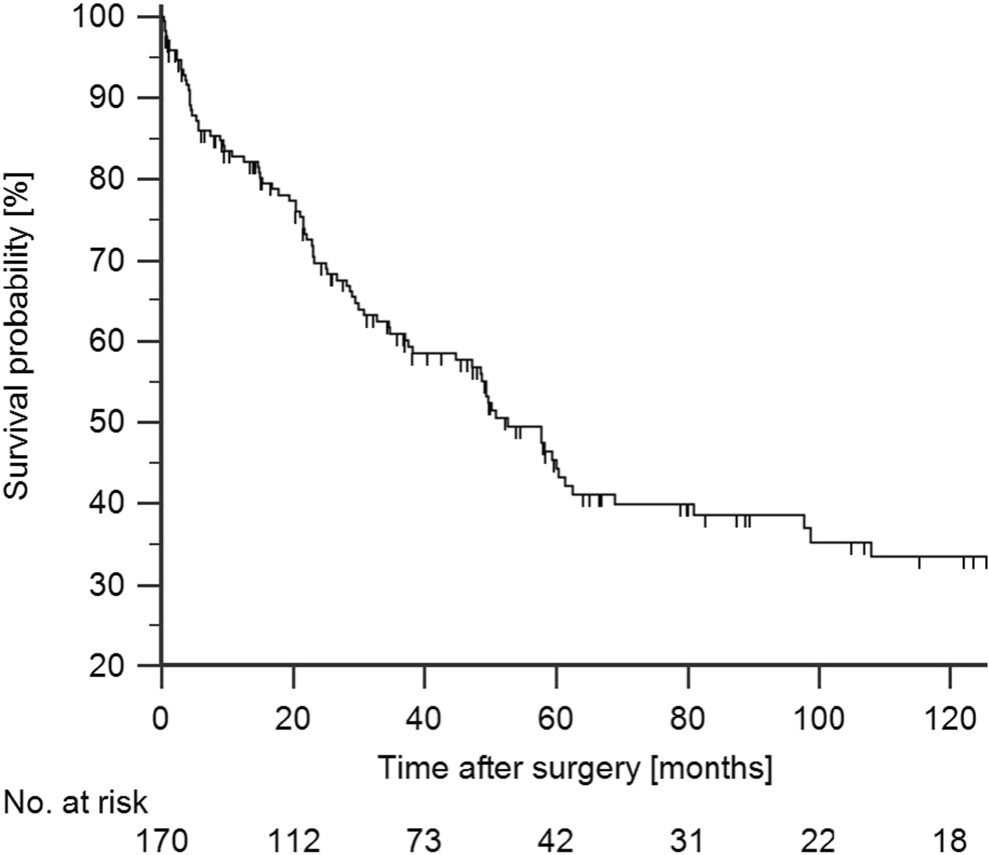
Overall survival in 170 patients undergoing resection of pathology proven PHC in the AMC. The 5-year survival rate after resection was 44.3%

The median survival after resection of confirmed malignancy was 52.6 months. The 5-year survival after resection was 44.3% (Fig. 3).

### Pitfalls of diagnosis

#### Differentiation between malignant and benign disease

**Fig. 4:**
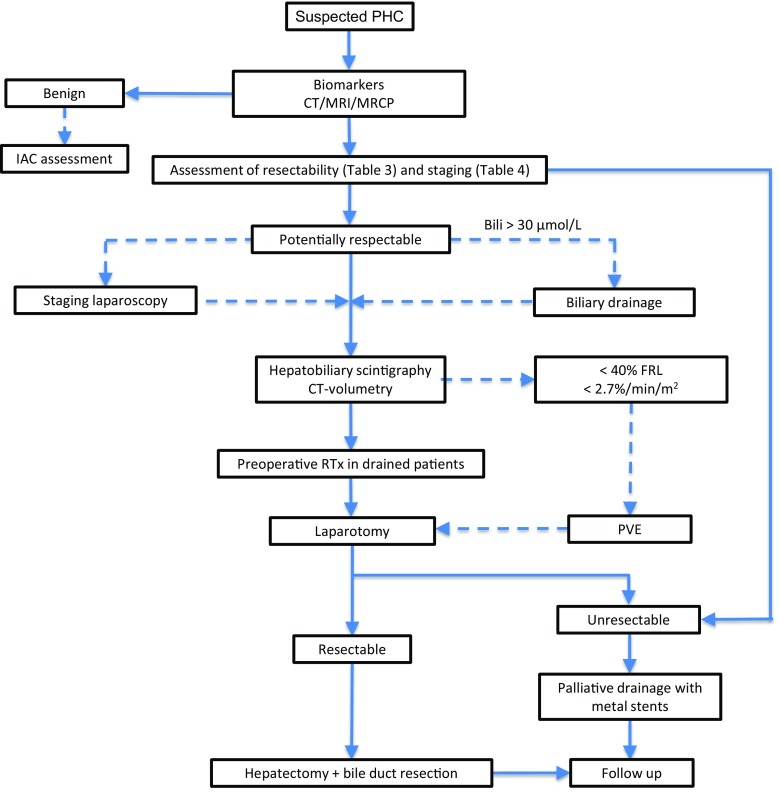
Flowchart showing work-up and treatment of patients suspected of PHC

In patients with a presumed PHC, it is highly desirable to obtain a definitive diagnosis (Fig. 4). Benign biliary tract strictures are difficult to differentiate from malignant disease [25–27]. In recent years, IgG4-associated cholangitis (IAC) has been identified as a disease entity that may mimic PHC, both clinically as on imaging studies. It belongs to the spectrum of IgG4-related disease, a systemic disease which can affect many other organs as well [28–30]. Of all resections for presumed PHC worldwide, 8–22% of patients turned out to have a benign disease on microscopical examination of the resection specimen [26].

##### Endoscopic techniques

Endoscopic retrograde cholangiopancreatography (ERCP) combined with brush-cytology for microscopical examination has been the standard diagnostic modality for years [31–33]. PHC, however, frequently shows a submucosal growth pattern resulting in a low sensitivity of brush cytology of 27–56% [31, 34, 35]. Various techniques have been investigated to increase sensitivity of cytological samples. These include fluorescence in situ hybridization (FISH) which is reported to increase sensitivity to 69–93% [36–38]. Mutation analysis has not been used widely, but seems mainly to increase specificity [39]. The use of stiffer bristles or repeated brushings also has not increased the diagnostic yield of brush cytology [40–42]. Other endoscopic techniques have emerged as well. The use of endobiliary forceps biopsy during ERCP resulted in a higher detection rate ranging from 44 to 89% [43, 44]. The technique however is challenging, especially in more proximal lesions as it is difficult to navigate and position the forceps. Consequently, it has not found wide application [44–46].

Cholangioscopy offers direct visualization of biliary strictures and seems to improve the diagnostic yield of routine cytology. Percutaneous cholangioscopy requires percutaneous biliary access and multiple dilatations to allow access of the cholangioscope. Single operator cholangioscopy (Spyglass, Boston scientific, Natick, MA, US) is introduced through a duodenoscope and is used in combination with Spybite Biopsy Forceps [47, 48]. Using these techniques, the sensitivity in diagnosis of biliary strictures has increased to 74.7% [49]. Cholangioscopy enables targeted biopsies increasing sensitivity and specificity to detect PHC to 66 and 97%, respectively, in a meta-analysis. Single operator cholangioscopy seems a useful new step in centers experienced with ERCP and brush cytology.

Alternatively, intraductal ultrasound (IDUS) enables detailed imaging of the bile ducts and periductal tissue. IDUS has been reported to improve diagnostic accuracy of ERCP from 58% to 90% [33, 50]. However, stents that are often required to drain obstructed bile ducts make the interpretation of IDUS difficult. If this is the case, the use of endoscopic ultrasonography (EUS) in combination with fine needle aspiration (FNA) may be preferable [50–52]. These techniques however, require specific expertise to reach their maximum potential and their success rates must be partially attributed to the experience of their users.

##### Serum markers

The limited ability to reliably acquire tissue samples has resulted in an ongoing quest for serum biomarkers. The additional use of serum markers to distinguish IAC from PHC has been an area of extensive research. Serum IgG4 levels (ULN = 1.4 mg/ml) have limited diagnostic value when only slightly increased, since up to 15% of patients with PHC have elevated sIgG4 levels as well [53, 54]. Recently a new test has been developed measuring the IgG4/IgG RNA ratio. This test distinguishes IAC accurately (94% sensitivity, 99% specificity) from PHC and primary sclerosing cholangitis [55]. The value of this test awaits further clinical assessment.

Biomarkers are also needed to monitor patients with an increased risk of PHC such as in primary sclerosing cholangitis [56]. The conventional serum markers CA19–9 and CEA are frequently used in gastrointestinal malignancies. However, the diagnostic value of CA19–9 is debated because of its variable sensitivity of 33–93% and specificity of 67–98%. Its use as a prognostic biomarker seems more valuable [57–60]. Furthermore, CA19–9 may be elevated in benign biliary disease and/or in the presence of cholestasis, impairing its use as a reliable biomarker especially in biliary tumors [61]. The same applies for CEA with a sensitivity of 33–84% and specificity of 50–88% [58] in pancreato-biliary malignancies.

### Staging and resectability

#### Criteria for the assessment of resectability

Initial imaging is crucial in establishing diagnosis and in determining whether a patient is a candidate for resection. The goal of curative resection is to achieve negative margins (R0) while preserving sufficient volume and function of remnant liver with adequate portal venous and hepatic arterial blood supply. Factors to consider to determine resectability are included in Table 3 [62].

**Table 3:** Criteria for the assessment of resectability in PHC

Criteria for the assessment of resectability
Presence of (extra) hepatic metastases
Presence of lymph node metastases confined to hepatoduodenal ligament (N1) or lymph node metastases along the common hepatic artery and/or celiac axis (N2)
Possibility of achieving free ductal margins on the side of the FRL
Involvement of portal vein bifurcation
Involvement of hepatic artery branches
Volume and function of FRL

Unresectability can result from either extensive local disease (including vascular and nodal involvement), presence of distant metastases or comorbidity of the patient. Local unresectability can be due to involvement of the portal vein and hepatic artery on the side of the future remnant liver without the possibility of a vascular reconstruction, extensive bilateral proximal infiltration of the tumor into secondary biliary radicles (segmental bile ducts) and/or massive extension of tumor into the liver parenchyma. Furthermore, extrahepatic metastases including distant lymph node metastases beyond the hepatoduodenal ligament (N2 nodes) are associated with poor survival and in most centers, are considered as unresectable as well.

It should be emphasized that local resectability depends on biliary anatomy at the liver hilum. The hepatic duct confluence is defined by the convergence of the right and left hepatic ducts, at which site many anatomic variations exist [63]. In 20% of cases, the anterior and posterior sectorial branches of the right ductal system drain directly into the main hepatic duct. This may give rise to confusion as when a hilar tumor involving the right anterior and posterior sectorial branches in these cases is combined with segmental involvement on the left side, the tumor is defined by the Bismuth-Corlette classification as type IV, which in many textbooks is considered unresectable. A type IV tumor in this situation however does not preclude a radical resection using an extended left hepatectomy. The same holds true for a tumor extending into the right sectorial ducts involving a low inserting segment 4 duct of the left biliary system. Although defined as Bismuth-Corlette type IV, this tumor can of course be radically resected using an extended right hemihepatectomy. Resectability depends on hilar biliary anatomy and it is therefore important that resectability is assessed by hepatobiliary surgeons with expertise in PHC [64].

#### Imaging

Imaging plays a decisive role in the diagnosis, staging and assessment of resectability. PHC manifests with various morphological growth patterns that can be recognized on imaging to enhance the diagnostic confidence, determine management and to provide additional information on prognosis. However, imaging can also lead to confusion due to overlapping appearances with other hepatobiliary diseases, including benign lesions. Important conditions to consider are other causes of biliary dilatation such as choledocholithiasis, PSC, IAC and biliary dilatation due to centrally located colorectal metastases [65]. Ultrasound is usually the initial test to evaluate patients with suspected bile duct obstruction [66, 67], and may provide information on the level of obstruction in the biliary tree.

##### Cross-sectional studies

CT and MRI are commonly used in various combinations with cholangiographic studies, in the diagnosis and preoperative planning of PHC. CT with iv contrast offers the opportunity to assess full extension of the tumor in detail and determine resectability [68, 69]. If PHC is suspected, imaging is preferably performed before stenting for biliary drainage, since the images will be obscured by the plastic or metal stent. In general, PHC may be recognized by dilated bile ducts, lack of communication between the left and right first-order bile ducts, crowding of bile ducts, ductal wall thickening and enhancement, and lobar atrophy. In some cases, a solid (mass forming) or papillary mass (intraductal growth type) may be seen.

The early arterial and late portal venous phases of a CT-scan aid to assess the relationship between tumor and (branches of) the hepatic artery and portal vein, which is important in determining resectability [70, 71]. Key elements for staging in imaging are defined in Table 4. According to a meta-analysis by Ruys et al., sensitivity and specificity of CT were 89 and 92% for assessment of portal vein involvement (encasement or occlusion are strong evidence), 84 and 93% for hepatic artery involvement and 61 and 88% for lymph node metastases, respectively [68].

**Table 4:** Key elements for staging of PHC

Key elements necessary for staging PHC
Location of primary tumor
Intra- or extrahepatic
Proximal common hepatic duct
Confluence of the left and right hepatic duct
Left or right hepatic duct
Intraductal growth type
Local extension
Segmental duct involvement (including Bismuth-Corlette classification)
Mentioning biliary variant anatomy
Vascular involvement (portal vein and/or hepatic arteries, including vascular variations and presence of stenosis of celiac axis or mesenteric artery)
Lymph nodes
Regional N1; cystic duct, common bile duct, proper hepatic artery and portal vein nodes
Metastatic N2; common hepatic artery, periaortic, pericaval, superior mesenteric or celiac artery nodes
Distant metastasis
Noncontiguous liver, peritoneum, bone, other

MRI with iv contrast provides an acceptable alternative to CT in the evaluation of PHC. Both CT and MRI have similar staging accuracy, including that of nodal staging [72]. The advantage of MRI is that combined with cholangiography (MRC), it provides anatomical definition of the biliary tree. Whether CT or MRI is used should be based on local expertise and accessibility to one of these modalities [73].

[^18^F]-FDG PET-CT has no additional value in the diagnosis and staging of PHC. In the hilar area, it is difficult to distinguish tumor from concomitant inflammation. Furthermore, for the identification of nodal involvement, [^18^F]-FDG PET-CT has a sensitivity and specificity of 67 and 68%, respectively [74, 75]. Hence, it does not provide additional diagnostic yield in comparison with CT.

##### Cholangiography

MR cholangiography (MRC) combined with MRI has comparable staging accuracy with that of CT combined with direct cholangiography [72]. Alternatively, direct cholangiography using ERCP or percutaneous transhepatic cholangiography (PTC) can also be used. PTC may be more helpful in assessing the extent of proximal tumor infiltration. ERCP can also be combined with cytological or tissue sampling, albeit sensitivity and specificity are low (see above). A major disadvantage of direct cholangiography is its invasiveness, including the risk of inducing infection, pancreatitis, bleeding, inflammation and pain. Direct cholangiography for diagnostic purposes is therefore, only rarely performed. Especially ERCP entails retrograde contamination of the obstructed bile ducts with increased risk of cholangitis. Subsequent drainage of the visualized bile ducts using one or more stents is therefore mandatory. ERCP and PTC are preferably used for therapeutic purposes to drain the obstructed bile ducts in the palliative setting or preoperatively, to prepare the patient for resection. In the latter situation, the aim is to drain the biliary system of the future remnant liver while leaving the part to be resected alone.

### Staging systems

There are many factors associated with resectability, prognosis and prediction of long and short-term survival after resection of PHC [15, 76–79]*.* The most commonly used staging systems include the American Joint Committee on Cancer (AJCC) staging system with incorporated TNM classification, the Bismuth-Corlette system, the Blumgart T-staging system (MSKCC classification) and a classification recently proposed by the International Cholangiocarcinoma Group for the staging of PHC [14, 15, 20, 77, 79–81]. The AJCC staging system is based on pathology assessment of the resection specimen and is mainly used postoperatively as a prognostic tool. The Bismuth-Corlette classification system, introduced in 1975, is used to describe proximal involvement of tumor into the bile ducts [4]. This system is mainly informative to surgeons for planning of the type of resection, but does not determine resectability since other parameters such as distant metastases and vascular involvement are not included. The Blumgart classification system takes in addition to bile duct involvement, portal vein involvement and lobar atrophy into account as well [82]. However, since its introduction in 1998, the indications for (extended) resections have expanded rendering the Blumgart system now less applicable. The classification system proposed by the International Cholangiocarcinoma Group for the Staging of PHC takes into account most of the variables used in the previous systems: suspicious lymph nodes, extent of bile duct involvement, extent of vascular involvement, suspected tumor size and lobar atrophy. As in the other systems, the information is largely descriptive [83].

The staging systems used to date are mainly surgery oriented. Each has its merits, but all are limited to the anatomical description of the tumor and are therefore limited in their ability to predict the likelihood of an R0 resection. Furthermore staging systems have been criticized for having poor predictable quality in different populations [20, 79, 84]. Ideally, a staging system would preoperatively predict the likelihood of resectable disease along with as well, prognostic value.

### Staging laparoscopy

For optimal determination of resectability, patients with potentially resectable PHC may undergo staging laparoscopy to detect the presence of occult tumor manifestations. Staging laparoscopy may detect small liver and/or peritoneal metastases that are undetectable on routine imaging avoiding a futile laparotomy [84–86, 155]. A thorough inspection of the liver, gallbladder, hepatoduodenal ligamen*t* and peritoneum is undertaken. The lesser sac is routinely opened and the common hepatic artery is examined, lymph node station 8 (N2) is then identified and biopsied for pathological evaluation. All other suspicious lesions, based on intraoperative inspection or previous imaging, are biopsied for histopathological analysis. Although not widely used, the combination with laparoscopic ultrasound has been reported to increase the yield of the staging procedure to some extent. In a meta-analysis by Coelen et al., which included 832 potentially resectable PHC patients, a pooled sensitivity of 52.2% was found to detect unresectability [14]. Based on our own experience in 273 patients undergoing staging laparoscopy for PHC, we developed a risk score that estimates the chance of unresectability. This risk score includes the following factors: tumor size, portal vein involvement, suspected lymph-node metastases and suspected (extra) hepatic metastases. It showed good discrimination between resectable and unresectable disease (AUC 0.77, 0.68–0.86 95% CI) [16].

### Assessment of future remnant liver

#### Liver volumetry

Since extended liver resections are often required, it is critical to assess the FRL preoperatively where CT-volumetric analysis is the standard technique. The segments of the FRL are delineated on the CT images and the ratio of the remnant liver and the total liver, with subtraction of tumor volume is calculated. This delineation technique gives an indirect measurement of the liver function [87, 88].

It is assumed that a FRL-volume of > 25–30% is considered a safe cutoff for patients with healthy liver parenchyma, whereas > 40% is used in patients with compromised liver, like in patients with (post)cholestatic liver that is damaged by longstanding biliary obstruction and possible cholangitis [88–90]. In literature the acceptable minimum volume of FRL in regard with parenchymal disease is variable and controversial (10–40%) [91–93]. In PHC, a FRL volume of more than 40% is usually considered. A disadvantage of FRL volumetry is that individual patient characteristics are not taken into account and that the delineating technique is prone to error [94, 95]. Especially in patients with compromised liver, discrepancies have been reported between CT volumetry and postoperative outcomes [96] because the quality of the liver parenchyma is not taken into consideration [87, 97].

#### Liver functional tests

Because liver volume does not equal liver function and function is not homogeneously distributed in the liver [97], we rely more on assessment of the function of the FRL, rather than on volume alone. ^99m^Tc-mebrofenin hepatobiliary scintigraphy (HBS) is a validated quantitative dynamic liver function test for which mebrofenin, an iminodiacetic (IDA) derivate, is used as a tracer. This agent is mainly taken up by the hepatocytes and is subsequently excreted in the bile without undergoing any biotransformation. The hepatic uptake is mediated by the same transport mechanisms as that of various endo- and exogenous substances, making it an ideal agent to assess liver function. HBS consists of an early dynamic phase, acquired directly after intravenous injection of mebrofenin, during which the mebrofenin uptake rate (MUR, %/min) is measured [98]. This corresponds with the total liver function. Immediately afterwards, a SPECT acquisition combined with low-dose CT is made, falling in the period in which mebrofenin is accumulated in the liver. The SPECT data provide information on three-dimensional, segmental distribution of function. The low-dose CT is solely used for attenuation correction and anatomical mapping [99].

The FRL is delineated on the SPECT images for calculation of the functional share (%). This is then multiplied by the total liver function (MUR) to calculate function of specifically the FRL. This method provides visual and quantitative information on regional liver function. Functional share of the FRL is corrected for body surface area (BSA, m^2^) using the Mosteller formula, to individualize the results based on the individual metabolic needs [100]. The current cutoff for a safe resection is a FRL function of at least 2.7%/min/m^2^ [97].

HBS can be used in patients with normal or impaired quality of liver parenchyma alike using the same cutoff value. MUR has been shown to correlate well with ICG clearance [101]. A limitation of using HBS in patients with PHC is that the uptake of bilirubin is competitive with mebrofenin as both are taken up by the same hepatocyte transporters [102]. In these patients, hepatocyte function is likely to be decreased which will be reflected by HBS, with additional underestimation due to competition. These receptors are downregulated during hyperbilirubinemia, but their expression gradually normalizes after drainage [103]. Considering this interaction, HBS should not be performed in patients with high bilirubin levels (> 30 μmol/L) and is usually postponed until adequate biliary drainage has been achieved [104].

### Preoperative preparation of the patient

#### Obstructive jaundice and biliary drainage

Patients with PHC usually present with obstructive jaundice. This phenomenon has a negative effect on liver function, increases the risk of biliary infection and impairs cellular immunity [105]. Preoperative biliary drainage is used to create a safer environment prior to liver surgery. It reduces jaundice, improves liver function and the patient’s condition, at the same time improving the ability of the liver to regenerate postoperatively [85, 105–109]. The impact of these effects is particularly high in patients with an insufficient FLR and preoperative drainage has shown to improve outcomes especially in patients requiring extended resections [108]. On the other hand, drainage-related complications may be severe and it is therefore advisable to only drain patients with a substantially increased bilirubin and small FLR [85, 110]. Drainage-related complications such as cholangitis may severely deteriorate a patient’s condition and increase the risk of postoperative morbidity and mortality [50, 85, 105, 111]. Preoperative cholangitis is caused by contamination of the biliary tract during drainage procedures. It is therefore advisable to give prophylactic antibiotics previous to any drainage procedure [3, 23, 107, 112]. Any episode of cholangitis induced after biliary decompression should be treated with antibiotics and additional drainage or drain revision if necessary [21]. Refractory cholangitis is often caused by incomplete biliary drainage and requires adequate endoscopic or percutaneous stenting of residual, obstructed parts of the biliary tract. Patients should not undergo surgery earlier than that they have fully recovered from cholangitis [21].

The optimal drainage method is still a much-debated topic, in which surgeons tend to favor the percutaneous approach for reasons of direct access to the biliary duct and postoperative use of the intraluminal drain(s) across the hepaticojejunostomy.

Although percutaneous biliary drainage (PTBD) seems associated with higher postoperative morbidity, further prospective studies are needed to better define the optimal mode of biliary drainage in PHC [112, 113]. Furthermore, PTBD might be complicated by portal vein thrombosis or seeding metastasis that may change resectability of the tumor [110, 114]. For now, endoscopic biliary drainage (EBD) is still the preferred method in most Western countries.

Endo-nasobilairy drainage (ENBD) is the advocated method in many Asian countries. As in PTBD, it provides more precise information on the extent of cancer along the bile ducts [115]. Some authors reported less complications and a high success rate of ENBD compared to EBD [116, 117]. However, others reported comparable results with EBD and PTBD [110]. Western centers generally do not perform ENBD because the nasal tubes easily dislocate and from the patients’ perspective, are usually less well tolerated. ENBD drains bile externally via the naso-gastro-duodenal tubes, precluding bile entering the intestinal system and therefore demands bile suppletion. This is then only possible via the oral route or a second gastroduodenal tube.

Several retrospective studies have been performed concerning the optimal drainage method [118] mainly emphasizing that each method comes with its own set of complications such as cholangitis, pancreatitis or vascular complications. Until evidence has been presented, EBD remains the reference method in most Western countries [107, 109].

The balance between the benefits and risks of biliary drainage is fragile and drainage strategies should be optimized in order to minimalize the risk of intrinsic complications. Due to these risks, it may be advisable to undertake surgery without prior drainage provided there is a surplus of remnant liver volume. Wiggers et al. showed that with a FLR > 50% preoperative biliary drainage was of no added value [88].

Hence, in patients requiring extended resection, we now use selective preoperative biliary drainage of only the future remnant liver when FRL is small (< 50%) whereas complete preoperative biliary drainage is mandatory in the event of (recent) cholangitis.

#### Portal vein embolization

If the FRL has not sufficient volume or function to undergo a safe resection, portal vein embolization (PVE) is the standard intervention to increase the functional capacity of the FRL. The local hemodynamic changes proposedly result in a release of a range of interleukins and growth factors that induce hypertrophy of the non-embolized lobe. In our cohort of PHC patients, the cutoff for proceeding with PVE is a FRL volume of less than 40% and/or function less than 2.7%/min/m^2^. In the absence of cholangitis, the biliary system of the embolized lobe needs not be drained, since unilateral cholestasis may even have a synergistic effect on the hypertrophy response of the non-embolized lobe. After a period of 3 weeks after PVE, CT and HBS are repeated and reassessed. We have shown that functional increase occurs more rapidly than volume, suggesting a shorter waiting time until resection can take place [119].

In the series reported by the Nagoya group, PVE showed to improve the surgical outcomes of PHC [10]. PVE is considered a safe procedure with an overall morbidity rate of 2.2%. Most common complications are hematoma, hemobilia, septic complications, backflow of embolization material and thrombosis in the FRL [120]. Olthof et al. analyzed the incidence of postoperative liver failure in a combined series of two Western centers specialized in PHC. A risk score was proposed to select candidates for PVE based on FRL volume combined with jaundice at presentation, preoperative cholangitis and preoperative bilirubin level > 50 μmol/L [121].

Accelerated tumor growth due to PVE does not seem to influence the survival of PHC patients [122, 123]. PVE however, does predetermine the side of the resection and in case of new findings that may require a change of strategy, this cannot be reversed. If the patient becomes unresectable due to disease progression in the waiting time, the atrophy-hypertrophy reaction stabilizes with time and the overall liver volume and function remain unchanged. However, the persistence of the atrophied, usually contaminated cholestatic liver lobe can be accompanied with adverse effects such as liver abscess, complicating further palliative treatment of the patient who typically will need repeated treatment with biliary stents [124].

Additional embolization of segment 4 in preparation of extended right hemihepatectomy is an option depending on the target increase of FRL volume that needs to be attained. To this end, the left portal vein branches to segment 4 are occluded along with embolization of the right portal venous system. The technique is challenging and requires an experienced interventional radiologist since access to the left portal venous system can give additional risk of injury. Backflow of embolization material into the left portal venous system can lead to inadvertent embolization and thrombosis of the portal veins supplying the FRL. Alternatively, to decrease these risks, partial embolization of only segment 4a can be performed [120, 156].

#### Preoperative radiotherapy

There is no general consensus regarding the use of neoadjuvant therapy for PHC. Low-dose preoperative radiotherapy (3 × 3.5 Gy in 3 days prior to resection) was instituted in our center in patients with resectable PHC who received preoperative drainage to prevent seeding metastases. The increased risk of seeding metastases after biliary drainage is an area of debate [114, 125] with various outcomes reported in literature [86, 126]. In our cohort, seeding metastasis in up to 20% of patients after endoscopic stenting has been observed in the laparotomy scar or drain tract [86] . This complication was associated with tumor cells contained in the bile that inevitably contaminated the operative field after bile duct transection in the course of resection. After using preoperative radiation, no catheter tract recurrences after drainage have been reported [125]. However, there is no evidence for this concept that is uniquely applied in our center [127]. A recent study conducted in two Western specialized centers, did not show an association of seeding metastases with center or mode of preoperative drainage, i.e. endoscopic or percutaneous drainage [125]. New prospective studies are needed to develop guidelines on this topic.

### Surgical aspects

#### General considerations

**Fig. 5:**
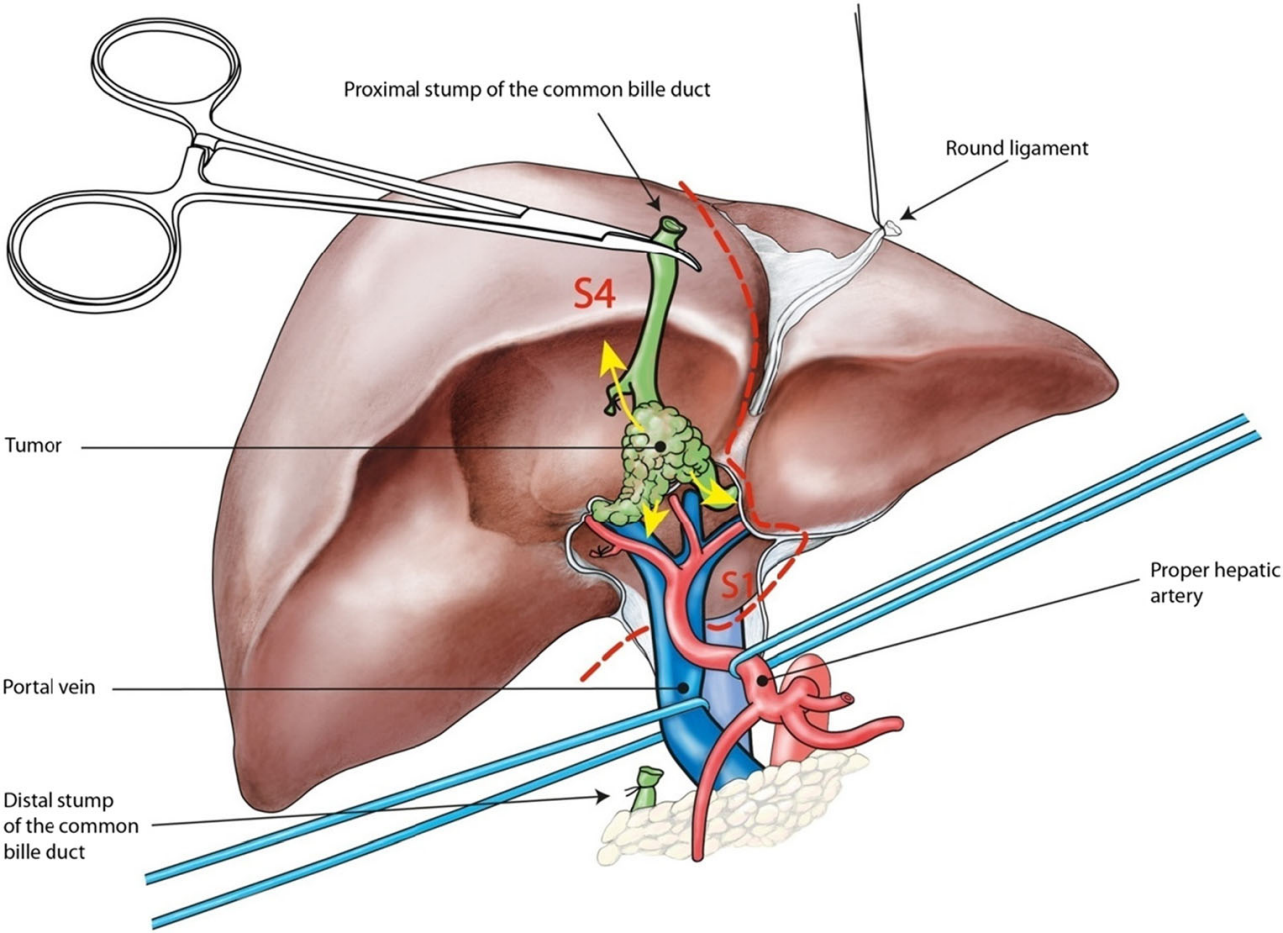
Extended resection for PHC should include the central sector (segment 4) with segment 1 along the antero-posterior axis of the liver. Depending on the predominant side of the tumor, a left (extended) or right extended hemihepatectomy is chosen for *en bloc* resection of the hilar area

The goal of surgical treatment is to achieve an R0 resection of the tumor along with clearance of the regional lymph nodes. Because of the central location of the tumor at the liver hilum and its proximal extension into the segmental bile ducts, complete resection requires excision of the extrahepatic biliary duct in combination with extended liver resection. The close relation of the tumor with the right and left portal vein and the hepatic artery branches often demands concomitant vascular resections and reconstruction. The Japanese surgeons were the first to show in the nineties of the previous century, that this aggressive approach resulted in improved long-term survival [128]. Still following these lines, radical resection entails excision of the liver hilum with (extended) hemihepatectomy including segment IV and the caudate lobe, complete lymphadenectomy of the hepatoduodenal ligament and excision of the portal vein bifurcation when involved [129]. Additionally, arterial resections are undertaken in order to achieve an R0 resection (Fig. 5).

The type of resection depends on location of the tumor and biliary anatomy at the hepatic duct confluence, the radial and longitudinal extent of tumor into the intrahepatic bile ducts and its association with adjacent periductal structures, portal vein and hepatic arteries. When viewing cross-sectional imaging studies, it is important to perceive the tumor in a three-dimensional fashion. The tumor extends along the right and left biliary ducts into the liver and at the same time, in anterior and posterior direction into the ducts of segment 4 and segment 1, respectively. Complete resection therefore should include the central sector of the liver along the antero-posterior axis including the segments 1 and 4 [62]. As the central sector anatomically is part of the left hemi-liver, there is an advantage of a left-sided approach comprising an anatomical left hemihepatectomy, whereas coming from the right, an extended right hemihepatectomy is required to include the central sector.

Intraoperative frozen-section pathological examination of the resection margins of the biliary ducts is performed to confirm radicality at the ductal level. In case of residual tumor in the resection margin, the level of biliary resection is extended although in our series, survival was worse in these patients compared to patients who had an initial free margin [108]. Some surgeons routinely drain the biliary ducts of the remnant liver after reconstruction using trans-anastomotic tubes. We usually do not internally drain the hepaticojejunostomy but when there are PTC drains in place, these are positioned across the anastomoses allowing access for possible postoperative cholangiography. As recently reported, leaving the drains open is not advised since the loss of bile negatively influences postoperative regeneration of the liver remnant [130].

#### Resection of the portal vein bifurcation: unconditional or on demand

Controversy exists regarding the unconditional, simultaneous *en bloc* resection of the portal vein bifurcation with the tumor. The hilar vessels run adjacent to the tumor with less than 1 mm between tumor and portal vein while perineural infiltration of the tumor along the bile ducts is a common feature of PHC. Because of this anatomical proximity, a no-touch technique was proposed by the Berlin group in which resection includes unconditional excision of the portal vein bifurcation *en bloc* with tumor excision and hepatectomy. Using this oncological strategy, dissection of the hepatic hilum is avoided and the risk of dissemination of tumor cells minimized. This technique however is less feasible in patients requiring left hepatectomies due to the fact that the right hepatic artery usually crosses the hepatic hilum directly anterior or posterior of the tumor, unless there is a displaced right hepatic artery originating from the superior mesenteric artery that runs along the right-lateral margin of the hepatoduodenal ligament. Also, reconstruction of the right portal branches with the main stem is technically more demanding.

Multivariate analysis of the Berlin series of resected PHC showed that portal vein resection was the only significant factor to influence patient survival after confirmed R0 resection. The 5-year survival rate of curative liver resection in their series was 65% with portal vein resection as compared to 28% without [131]. Other authors advise to only perform portal vein resection when during exploration, the portal venous bifurcation/contralateral portal venous branch is found to be invaded by tumor.

In our center, we do not advocate the unconditional excision of the portal vein bifurcation, also because portal venous reconstruction has been associated with an increased postoperative morbidity rate [88]. Modern preoperative imaging techniques now accurately demonstrate portal venous involvement. Relying on this information, we only resect the portal vein bifurcation *en bloc* with the tumor right away when there is evidence of vascular involvement on CT-scan. Otherwise, the decision to resect the portal vein bifurcation is made intraoperatively. This policy has led to portal vein resection in approximately 20% of our cases (Table 2). Survival analysis of our series showed an overall 5-year survival of 44.3%, which is comparable with the 5-year survival of 43% reported by the Berlin group in a series of patients that underwent R0 resection using unconditional *en bloc* portal vein resection [132].

Concomitant resection of the portal vein bifurcation with extended hemihepatectomy is followed by end-to-end anastomosis of the left portal vein with the main portal venous stem. Complete mobilization of the left portal vein by detaching all side-branches to segments 4 and 1 facilitates reconstruction. As the caliber of the left portal vein usually is much smaller, it is important to bevel the anastomosis after oblique clamping in order to prevent stenosis of the anastomosis. With (extended) left hepatectomy, reconstruction of the right portal branches with the main stem is technically more demanding.

#### Concomitant resection of the hepatic artery branch feeding the remnant liver

Adequate arterial perfusion is crucial to function of the remnant liver. The left hepatic artery runs along the medial side of the hepatoduodenal ligament and is therefore less at risk for tumor involvement. The right hepatic artery with its right anterior and posterior branches however, is frequently infiltrated by tumor. The choice of performing a right or left resection is often dictated by the side of the liver in which the hepatic artery branches are free. In PHC predominantly involving the left liver, (extended) left hemihepatectomy with concomitant resection of the right hepatic artery is hampered by preserving a tumor free, intrahepatic distal stump for arterial reconstruction especially when tumor mass is substantial. Microsurgical techniques are usually applied to create a safe anastomosis with the right posterior branch of the right hepatic artery in these cases. Combined arterial resection and reconstruction with portal vein resection is controversial. In the Nagoya cohort of resected patients, this challenging technique was associated with a mortality of 2% and a 5-year survival of 30% for patients with advanced cholangiocarcinoma [133].

Preoperative embolization of the branch of the hepatic artery feeding the future remnant liver and subsequent excision is not attractive in our view, because the biliary anastomosis depends on arterial periductal arterial perfusion and loss of arterial blood supply will lead to failure of the hepaticojejunostomies. Arterialization of the portal vein can be used as last resort when arterial perfusion of the remnant liver is sacrificed, however is preferably applied for salvage of the remnant liver [134].

#### Concomitant resection of segment 1

As pointed out above, the caudate lobe is part of the central antero-posterior axis of the liver and is preferably resected *en bloc* with the tumor and liver hilum. Although the segment 1 bile ducts often drain into the left ductal system, they may drain into any part of the hepatic duct confluence and these ducts are frequently involved by tumor as well. Routine S1 resection *en bloc* with (extended) hemihepatectomy has therefore been implemented at our institution since 1998 and has increased the rate of R0 resections and has resulted in improved survival [135]. *En bloc* excision of segment 1 is therefore recommended with resection of PHC.

#### Right or left (extended) hepatectomy

The decision to perform a right or left hepatectomy depends on local tumor extension, portal venous and hepatic arterial involvement and the FRL volume and function. For optimal preoperative preparation of the patient, such as biliary drainage or the need for PVE, it is important to preoperatively determine the side of the liver to be resected.

If the tumor extent and FRL volume allow both options, a right-sided (extended) hepatectomy is often preferred because it is more likely to achieve oncological radicality and is more a straightforward procedure for several reasons [136]. Firstly, because the biliary confluence is located on the right side of the hepatoduodenal ligament, a right hepatectomy allows more complete resection of the tumor. Also, the right hepatic duct is often short (< 1 cm) or even absent in case of a triple hepatic confluence, while the left hepatic duct has a relatively long and straight course until reaching the border of the left portal vein and branching off into the ducts of segments 2 and 3 [137]. Therefore, tumors that invade the right sectoral ducts and even the segmental ducts to segment 4 (Bismuth-Corlette type IIIa-IV) can be radically resected by extended right hemihepatectomy. A disadvantage of a right-sided approach is that segments 2 and 3 are small and that in many patients preoperative right PVE is necessary before undertaking extended right hemihepatectomy.

Tumors predominantly involving the left ductal system (Bismuth-Corlette type IIIb-IV) require a left-sided approach. The advantage of a left-sided resection is that the remnant liver, i.e. the right liver segments, usually has more volume and resection can be extended farther into the right liver. Of note, the volume of segments 6 + 7 usually exceeds that of segments 2 + 3 which may direct the choice of a right or left-sided approach. A formal extended left hemihepatectomy following the medial margin of the right hepatic vein is technically more difficult and depending on involvement of the segment 8 ducts, part of the anterior right sector may be preserved (see below). A down-side of a left resection is that construction of the (often multiple) biliary anastomoses may be more complex with a higher risk of biliary complications.

#### Parenchyma sparing liver resection

As volume and function of the remnant liver are the most critical factors for postoperative outcomes, parenchyma preserving techniques can be applied in selected cases. These techniques can be used as an alternative to PVE or in addition to PVE, in order to spare as much functional liver tissue as possible.

In right-sided tumors that require an extended right hemihepatectomy, the cranial part of segment 4 (i.e. 4a) may be preserved depending on the level of involvement of the segment 4 bile duct. Free margin of the cut segment 4 bile duct is checked using frozen-section pathological examination.

In case of a left-sided tumor, a modified extended left hemihepatectomy may be undertaken. Extending left resection to include segment 5, the adjacent part of segment 8 may be preserved. Whether this can be performed depends on the proximal extent of the tumor into the right segmental ducts, and the anatomy of the right sectoral ducts (B5/8 and B6/7) in relation to the right hepatic duct and hepatic duct confluence.

A pitfall of sparing portions of the central sectoral segments 4 and 8 is cutting off their portal venous and arterial blood supply by resection of the tumor. The central position of the tumor often requires sacrifice of the middle hepatic artery to segment 4 or the right-anterior portal vein and hepatic artery branches to segment 8 leading to parenchymal infarction.

Another possibility for parenchymal preservation is performing a central liver resection (mesohepatectomy) when the bile ducts of segments 6 and 7 and the left lateral segments 2 and 3 are not infiltrated by the tumor. This complex procedure includes resection of the central sectors of the liver including segments 4, 5 and 8. In these cases, multiple jejunal anastomoses with the remaining intrahepatic segmental bile ducts are required [129]. A formal central resection is only possible when the vascular structures supplying the left lateral segments as well as the right-posterior segments 6 and 7 are free of the tumor and can be preserved.

#### ALPPS

In situ split of the liver in combination with portal vein ligation (ALPPS) has been introduced as a method to induce rapid hypertrophy of the FRL. Because of the higher mortality and morbidity reported in the initial series of ALPPS, this method along with great interest has generated a heated discussion in the surgical community [138, 139]. The advantage of ALPPS is debated in extended right hemihepatectomy as compared to complete embolization of the right portal venous system including segment 4 as described above. Several authors have reported their results of ALPPS in patients with PHC. Due to stenting of the biliary system and ensuing cholangitis, patients were at increased risk of interstage morbidity and mortality [140, 141]. ALPPS for PHC demonstrated poor outcomes with 48% perioperative mortality in the ALPPS registry [140]. We therefore for now, do not recommend ALPPS for resection of PHC and rather consider PVE with selective embolization of the left portal vein branches to segment 4 for augmentation of FRL volume in patients requiring extended right hemihepatectomy.

#### The extent of lymphadenectomy

Standard lymphadenectomy includes resection of lymph nodes around the extrahepatic bile duct, the portal vein and hepatic artery, as well as the lymphatic channels and nerves contained in the hepatoduodenal ligament. The number of lymph nodes resected is also relevant as less than 4 lymph nodes evaluated in the specimen was identified as a poor prognostic factor for time to recurrence [142]. Lymph node metastases that are limited to the hepatic pedicle or the hepatoduodenal ligament (N1) are included in the field of resection, but those along the common hepatic artery and/or celiac axis (N2) are considered distant metastases. N2-disease has a poor prognosis and disease specific survival of patients with para-aortic lymph node metastasis was similar to M1 patients, suggesting that survival is not influenced by the extent of lymph node dissection, but rather by the presence of N2 disease [12, 78]. Therefore, we do not recommend routine lymphadenectomy beyond the hepatoduodenal ligament.

#### Complications

Postoperative morbidity and mortality of patients with PHC is significant. Reported mortality ranges from 5 to 18% even in high volume centers, and morbidity as high as 60–70%, with around 50% severe complications (Clavien-Dindo grade III or higher) [21]. Table 5 shows the incidence of the most common complications as reported in literature together with the complications recorded in our own series. Risks are particularly high in patients with tumors requiring an extended resection. Liver failure is a dreaded complication after extensive hepatectomy and is a major cause of mortality in patients with PHC [21, 148]. The risk of postoperative liver failure is increased due to the combination of intra-operative blood loss, a small FLR and cholestasis [21, 88, 121]. Reported liver failure ranges from 3 to 25% [31, 88, 143, 144]. Biliary leakage from either the hepaticojejunal anastomosis or the parenchymal dissection surface ranges from 6 to 29% [31, 88, 143]. Infections range from 23 to 66% and bleeding complications from 4 to 9% [15, 31, 88, 145, 146] (Table 5).

**Table 5:** Complications and reported incidence in a selection of literature reports including the AMC series

Complication type	Incidence literature	Incidence AMC
Liver failure	3–25% [31, 88, 143, 144]	19% (29/156*)
Biliary leakage	6–29% [31, 88, 143]	30% (47/156)
Bleeding	4–9% [15, 31, 88, 145, 146]	8% (13/156)
Multi organ failure	1–3% [32, 87, 135]	2% (3/156)
Infections	23–66% [15, 31, 88, 145, 146]	22% (35/156)
Mortality	5–17% [88, 143, 147, 157]	9% (18/201)

*Total cohort: *n* = 156, missing *n* = 45

## Discussion and future perspectives

The management of perihilar cholangiocarcinoma is complex and requires close multidisciplinary collaboration between hepatobiliary surgeons, endoscopists, radiologists, medical oncologists and pathologists. In this review, we provide a summary of the current diagnosis and work-up in the light of extended resection and elaborate on future perspectives.

Establishing the diagnosis of PHC is still one of the most challenging aspects of the diagnostic work-up. New diagnostic endoscopic techniques such as SpyGlass and endoscopic ultrasound enable more precise biopsies, resulting in increased sensitivity and specificity in diagnosing biliary strictures [33]. These techniques will likely decrease the rate of misdiagnoses and bring down the number of futile resections performed for benign disease. The currently used conventional tumor marker CA19–9 is not particularly sensitive or specific. A combination of different markers seems more useful in the diagnosis and follow-up of PHC. Biomarkers such as CYFRA21-1 and MUC-5 need to be evaluated in larger cohorts to assess its clinical value. Molecular techniques such as circulating miRNA’s and Tumor Educated Platelets (TEP) represent an exciting area with great promise in this field [149, 150]. For now, approximately 50% of patients with suspicion on PHC undergo resection without a confirmed tissue diagnosis.

CT-volumetry has traditionally been the golden standard for assessment of a sufficient FRL. However, not only the quantity but also the quality of the FRL is important while liver volume does not correlate with liver function. In our cohort, total and regional (segmental) liver function was preoperatively evaluated using HBS. This quantitative method allows measurement of FRL-function and can be used in patients with impaired quality of liver parenchyma using the same cutoff value. A limitation of using HBS in patients with PHC is that bilirubin induces competitive uptake with mebrofenin as both are taken up by the same hepatocyte transporters. In patients with obstructive cholestasis, HBS may underestimate liver function when the biliary system is not completely drained.

The additional value of staging laparoscopy in the future is questionable, considering the low yield and further improvements in accuracy of preoperative imaging techniques. Using a risk score allows to predict unresectable PHC at staging laparoscopy in order to make a more selective approach to staging laparoscopy.

Since patients with PHC typically present with obstructive jaundice, decompression of the biliary tract is a much-debated topic. For the past, few years it has become clear that drainage of the biliary tract comes with a serious risk of drainage-related complications. Since obstructive jaundice impairs liver regeneration, biliary drainage is still advised in case of a small FLR. The optimal drainage method has yet to be established. In The Netherlands the DRAINAGE trial is underway to evaluate outcomes of PTBD vs. EBD in resectable PHC [108, 151]. In this multi-center trial with an all-comers design, all patients with a presumed PHC and cholestasis are randomized to undergo PTBD or EBD. The study is powered for drainage-related complications and postoperative outcomes. For now, we use selective preoperative, endoscopic biliary drainage of only the future remnant liver when FRL is small (< 50%) unless mandated by cholangitis.

The most important prognostic factor for long-term survival of PHC is a margin negative resection of the hilar tumor. In experienced hands, even Bismuth-Corlette type IV tumors can be resected with curative intent. R0 resection requires an aggressive surgical approach encompassing hilar resection in combination with extended liver resection, frequently accompanied with vascular reconstructions. These extended resections are associated with higher morbidity and mortality rates than experienced in liver resections without bile duct resection, probably because of the sequelae of obstructive jaundice. Survival after resection is however favorable, with 5-year overall survival rates comparable with survival after extended liver resection for colorectal liver metastases.

PVE is a widely accepted interventional procedure to increase FRL volume and function before undertaking major liver resection. This method of liver augmentation is especially of benefit in patients with PHC who require extended liver resection in predamaged livers. We therefore advocate the liberal use of PVE in patients with PHC in whom the FRL is below 40% of total liver volume. It is important to note that to obtain the maximum hypertrophy effect of segments 2 and 3, the side-branches of the left portal vein to segment 4 can be embolized as well. Obviously, selective embolization of the segment 4 branches requires expertise of the interventional radiologist as available in specialized centers. Although the first successful case of ALPPS was reported by Schlitt in a patient with PHC, the use of ALPPS in PHC as alternative to PVE is not recommended because of the reported high morbidity and mortality of the procedure in this category of patients [152].

There are no established strategies regarding the use of neo-adjuvant therapies in PHC. The only exception is neo-adjuvant chemo-radiation therapy prior to liver transplantation in a highly selected group of patients [153, 154]. The idea of a short course of radiation preceding resection was to eradicate free floating tumor cells in the bile, thus preventing viable tumor cells of contaminating the peritoneal surface. There is however no evidence for this concept.

The challenge in the coming years is to reduce morbidity and mortality associated with extended resections for PHC. Optimizing preoperative workup is key to achieve improved outcomes after extended resections.

## Conclusion

The field of work-up in PHC is changing with the introduction of newer modalities that have emerged over the past few years. Upcoming diagnostic modalities and molecular techniques might help to decrease the rate of misdiagnosis of benign, inflammatory disease. Assessment of liver function with hepatobiliary scintigraphy provides better information on the FRL than volume alone. The selective use of staging laparoscopy is advisable to avoid futile laparotomies. In patients requiring extended resection, selective preoperative biliary drainage is mandatory in cholangitis and when FRL is small (< 50%). Preoperative PVE is used when FRL volume is less than 40% and optionally includes the left portal vein branches to segment 4. ALPPS as alternative to PVE is not recommended in PHC. N2 positive lymph nodes preclude long-term survival. The benefit of unconditional *en bloc* resection of the portal vein bifurcation is uncertain. Although still associated with considerable morbidity and mortality, an aggressive surgical approach encompassing extended liver resection including segment 1, regional lymphadenectomy and conditional portal venous resection offers the only chance for long-term survival.
